# The Role of Frailty in the Treatment of Locally Advanced Rectal Cancer

**DOI:** 10.3390/cancers16193287

**Published:** 2024-09-27

**Authors:** Grzegorz J. Stępień, Jakub Włodarczyk, Kasper Maryńczak, Mateusz Prusisz, Mateusz Porc, Marcin Włodarczyk, Anna Waśniewska-Włodarczyk, Łukasz Dziki

**Affiliations:** 1 Department of General and Oncological Surgery, Medical University of Lodz, 92-213 Lodz, Polandmarcin.wlodarczyk@umed.lodz.pl (M.W.); lukasz.dziki@umed.lodz.pl (Ł.D.); 2 Department of Otolaryngology, Polish Mother’s Memorial Hospital Research Institute, 93-338 Lodz, Poland; anna.wasniewska@umed.lodz.pl

**Keywords:** frail patients, locally advanced rectal cancer, elderly patients, chemoradiotherapy, frailty syndrome, watch-and-wait strategy

## Abstract

**Simple Summary:**

As the population ages, the prevalence of frailty syndrome increases, particularly among elderly patients with rectal cancer. Frail patients are more vulnerable to stressors and may struggle with the standard treatment for locally advanced rectal cancer, which typically includes chemoradiotherapy, surgery, and chemotherapy. While this approach shows good clinical results, it may not be suitable for all frail patients due to potential side effects. Since frail patients are underrepresented in clinical trials, this article highlights the need for new treatment strategies for them. Routine screening for frailty in hospitalized patients is recommended, and treatment should be planned by a multidisciplinary team, including specialists such as geriatricians and oncologists. Adapting guidelines and treatment plans for frail patients is crucial for improving outcomes in this growing population.

**Abstract:**

Owing to the gradual aging of today’s population, an increase in the prevalence of frailty syndrome has been noticed. This complex state of health, characterized by decreased resilience and tolerance with concurrent increased vulnerability to stressors and adverse health-related factors, has drawn researchers’ attention in recent years. Rectal cancer, which constitutes ~30% of all colorectal cancers, is a disease noticeably related to the elderly. In its locally advanced form, it is conventionally treated with trimodal therapy—neoadjuvant chemoradiotherapy followed by total mesorectal excision and adjuvant chemotherapy. Despite its good clinical outcomes and improvement in rectal cancer local control, as evidenced by clinical trials, it remains unclear if all frail patients benefit from that approach since it may be associated with adverse side effects that cannot be handled by them. As old patients, and frail ones even more noticeably, are poorly represented in the clinical trials describing outcomes of the standard treatment, this article aims to review the current knowledge on the trimodal therapy of rectal cancer with an emphasis on novel approaches to rectal cancer that can be implemented for frail patients.

## 1. Introduction

Nowadays, it is undoubtedly observed that the world population is aging. According to statistical predictions for the future, the proportion of people aged 65 years and over will be increasing [1,2]. With aging, there is a substantial growth in the prevalence of frailty syndrome among the population [3]. In the literature, multiple approaches to the concept of frailty were proposed; most commonly, this term has come to be used to refer to a multidimensional, complex state of health characterized by decreased resistance to various stressors and increased vulnerability of the body [4,5,6,7].

Colorectal cancer (CRC), regarding incidence, is the third most common malignancy worldwide [8]. Generally, oncological diseases are intrinsically associated with old age, and CRC is predominantly diagnosed in patients aged 65 years and over. In particular, rectal cancer accounts for ~30% of all colorectal malignancies, and interestingly, this proportion was shown to have gradually increased over recent decades [9].

Nowadays, there are a few treatment options that oncologic surgeons can propose to rectal cancer patients. Standardly, rectal cancer in its locally advanced stage is treated using neoadjuvant chemoradiotherapy (chemotherapy with concurrent radiotherapy) followed by total mesorectal excision and adjuvant chemotherapy [10,11,12]. Of course, not every patient is eligible for this therapy, especially frail patients who may generally present higher rates of complications and mortality [4,13].

Due to the common underrepresentation of elderly patients, and more acutely, frail patients, in clinical trials and prospective studies [14,15,16], to our best knowledge, comprehensive guidelines for treating rectal cancer in patients with diagnosed frailty syndrome are still lacking. The goal of this paper is to provide a review of the current state of knowledge of treatment methods, their complications, and outcomes in frail patients diagnosed with rectal cancer. Gaps in the knowledge and the literature may help to identify discrepancies and carry out new research in favor of patients with frailty.

The discussion is organized into four major sections. In Section 3, we summarize general information about frailty syndrome, its hallmarks, potential risks, and implications for patients. Section 4 focuses strictly on rectal cancer—we cite up-to-date statistics and results of the standard treatment approach in the general and elderly populations. In Section 5, we describe a novel approach to locally advanced rectal cancer (LARC) treatment, the “watch-and-wait” strategy, with an emphasis on older and frail patients. The last part of the discussion, Section 6, addresses the underrepresentation of frail patients in clinical trials and future research directions.

## 2. Research Methodology

The articles focused on the treatment, outcomes, and survival of frail patients diagnosed with rectal cancer were searched in PubMed, Mendeley, Google Scholar, Scopus, and Ovid databases by using these keywords in various combinations: “rectal cancer”, “rectum cancer”, “frail patients”, “frailty”, “geriatric”, “elderly patients”, “treatment”, “mesorectal excision”, “surgery”, “chemoradiotherapy”, and “watch-and-wait strategy”. Initially, 1981 records were identified based on the mentioned keywords. A total of 1867 papers were excluded after title or abstract screening. In total, 114 full-text articles were assessed for eligibility. Finally, 68 studies were selected to be discussed in the review. References cited in these papers were also included. We excluded conference abstracts, papers not in English, and those whose full-text manuscripts could not be obtained. In our view, the references that we chose are most relevant to the multidimensional subject matter of rectal cancer in frail patients.

## 3. Frailty Syndrome

In recent decades, a shift in attitude toward elderly patients, their age-related functional decline, and morbidity has been observed. Studies began to focus on inevitable biological changes in the human body associated with aging and its impact on survival and treatment outcomes in oncological diseases. The distinctiveness of old age was pointed out. Simultaneously, research articles started to bring awareness to the problem of frailty, the syndrome that is most commonly found in older patients [3].

Frailty syndrome was described in the literature in a multidimensional way, and several concepts have been proposed. Until then, there was no universal definition of frailty and no all-encompassing way to measure that [4,5,17]. Generally, researchers seem consistent that this complex syndrome is regarded as a state of decreased resilience, tolerance, and physiological reserves of the organism, with increased vulnerability to stressors and adverse endogenous and extrinsic factors [4,5,18,19,20,21]. There are some hypotheses regarding the basis of frailty syndrome. Studies linked this phenotype with a chronic proinflammatory state, mitochondrial dysfunction, and dysregulation of hormonal activity [1,18,22,23]. The characteristic of this syndrome is that a frail patient is regarded as not only somatically limited but also may have a mental illness, may be undernourished, and may experience social isolation [4,5]. Frailty also has an impact on the economy and health services since it is associated with additional costs of hospitalization [24].

Undoubtedly, frail patients are susceptible to adverse outcomes, treatment intolerance, longer hospital stays after surgery, and worse post-surgical recovery; they have a higher chance of falls and death [18,19,20,25,26,27,28]. Dasgupta et al. conducted a study on 125 patients aged 70 years or older who received elective, non-cardiac surgical procedures. The study found a significant association between the patient’s age and frailty, assessed through Edmonton Frail Scale (EFS) scores, and the likelihood of postoperative complications occurring. The odds ratio (OR) for age was 1.14 (95% CI, 1.05–1.24), and the OR for frailty was 1.22 (95% CI, 1.02–1.46). Additionally, age and EFS scores were independently linked to discharge to an institution and prolonged length of postoperative stay [25]. Moreover, the presence of frailty was described to have the above-mentioned outcomes in several studies [18,20,26].

**Figure 1:**
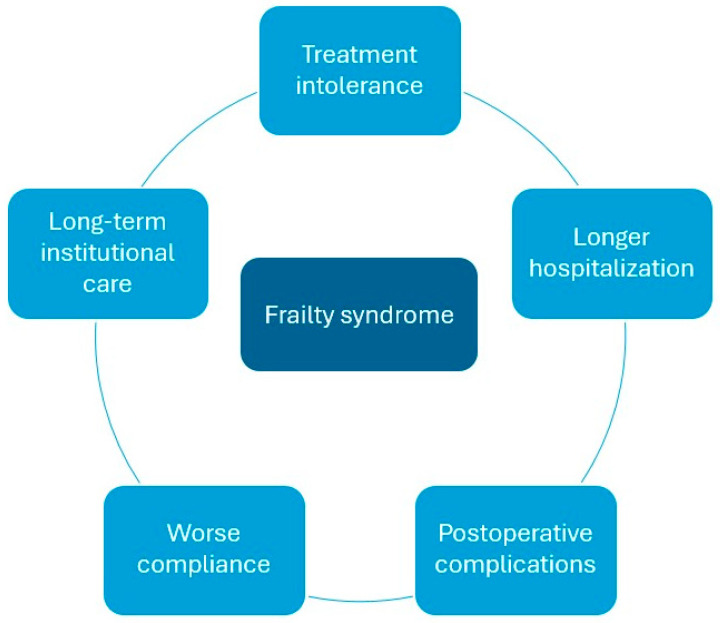
Possible consequences of frailty syndrome in rectal cancer patients.

Figure 1 summarizes the possible implications of frailty syndrome among rectal cancer patients.

The prevalence of frailty syndrome increases with advancing age. In the study of Gale et al., 5450 adults aged 60 and over were analyzed. The authors estimated frailty syndrome prevalence at 6.5% in participants aged 60–69 years and 65% in adults aged 90 years and above [3,29]. In terms of a heterogeneous group of cancer patients, the figures are even more noticeable. In the study of Handforth et al., in which 22 research articles concerning oncologic patients were evaluated, the median prevalence of frailty syndrome among this population was 42% (range 6–86%) [19].

As a result, not every older person is considered a frail patient; the overall condition of the elderly ranges from fit to frail. Moreover, this fact suggests that frailty is neither inevitable nor an equivalent of aging and comorbidity and can be potentially modified by medical interventions [1,4,19,21,30].

As frailty syndrome is not a permanent state, the implementation of frailty-assessing scales and questionnaires into daily medical practice seems a legitimate approach to identify patients who can benefit from more individualized in-hospital care and treatment strategies [1,21,22]. Such screening aims to identify patients who may not be suitable for standard treatment; thus, its associated risks and potential benefits (including quality of life) may be unacceptable for them [1,31]. Having such knowledge, it becomes possible to focus on the best outcomes for the patient. As an example of the efficacy and predictive value of screening among rectal cancer geriatric patients undergoing proctectomy, frailty measured by the simplified frailty index (sFI) was proven to be superior in predicting postoperative morbidity and mortality than older age alone [27]. In the study of Hall et al., the implementation of the Frailty Screening Initiative (FSI)—the preoperative screening of elective surgery patients utilizing the Risk Analysis Index (RAI)—was analyzed. A total of 9153 patients undergoing non-cardiac surgeries were included in the study. A total of 3878 of them were assessed by the FSI, and the rest (n = 5275) constituted a group of patients who were operated on before the introduction of the FSI. Patients identified as frail (RAI ≥ 21) were re-reviewed by a multidisciplinary team in terms of better decision-making and treatment optimization. As a result, implementation of the FSI was proven to decrease mortality at 30 (before FSI implementation vs. after FSI implementation—12.2% vs. 3.8%, *p* < 0.001), 180 (before FSI vs. after FSI—23.9% vs. 7.7%, *p* < 0.001), and 360 (before FSI vs. after FSI—34.5% vs. 11.7%, *p* < 0.001) days after surgical procedure. Interestingly, lower postoperative mortality was also spotted in the group of robust, non-frail patients [32].

Because of various approaches to the problem of frailty syndrome, plenty of diverse instruments for frailty assessment are described in the literature. The main limitation of frailty screening is that there does not exist an all-purpose tool, and the majority of them have been scantily evaluated in a series of studies concerning well-defined criteria and groups of patients [1,5,6]. There might be limitations associated with clinicians. In the study published by Saur et al., surgeons (almost half of them were working at academic centers) were asked to respond to a survey on their attitude toward elderly patients. In total, 48% of them perceived preoperative frailty assessment as mandatory, but only 6.4% were routinely evaluating their patients by using the CGA (Comprehensive Geriatric Assessment), and only 36.3% were collaborating with geriatricians [33]. Another possible limitation to routine screening may be the fact that frailty assessment is time-consuming, and there might be time and physician shortages in daily work [11,34].

Of course, further research is needed, but in our opinion, the available data on the impact of frailty may encourage practitioners to include frailty assessment as standard preoperative care in rectal cancer patients.

## 4. Rectal Cancer

### 4.1. General Information

According to the estimations for 2023, rectal cancer will account for 46,050 newly diagnosed cases in the United States. Still, it constitutes ~30% of all colorectal cancers. It is observed that it is mainly a disease of the elderly, with the majority of colorectal cancers diagnosed at the age of 65 years and over. The incidence rate increases gradually with age. However, in recent years, advances in chemotherapy, radiotherapy, and surgical management substantially have lowered mortality rates and increased 5-year survival rates [9].

The rectum is localized within the mesorectum, an area that is highly dense with lymphatic and vascular structures and vital organs. This location facilitates the tumor’s easy spread to surrounding tissues, and it quickly causes serious disturbances and health consequences [35,36]. Thus, a detailed assessment using MRI scans is needed for disease staging (invasion of the primary tumor and its size, and local lymph node involvement), the choosing of a surgical technique, and assessing the possibility of the tumor’s total resection [35,36,37,38].

### 4.2. Treatment of Locally Advanced Rectal Cancer

Locally advanced rectal cancer, clinically staged using MRI as T3-T4 and/or N1-2, is conventionally treated with a so-called “standard treatment” that involves neoadjuvant chemoradiotherapy (nCRT), followed by total mesorectal excision (TME) and adjuvant chemotherapy [10,39,40,41].

Sauer R et al., in their study concerning rectal cancer patients aged 75 years and below, made a comparison between preoperative and postoperative radiotherapy with concurrent fluorouracil-based chemotherapy that was given in conjunction with TME. The study demonstrated that the preoperative treatment regimen was significantly associated with sphincter preservation in the consecutive surgical procedure, more effective downstaging of the tumor, and a lower chance of local recurrences. The 5-year overall survival rate and the 5-year disease-free survival rate did not differ significantly in the neoadjuvant vs. adjuvant CRT regimen (76% vs. 74%, *p* = 0.80; 68% vs. 65%, *p* = 0.32; respectively) [42].

Neoadjuvant chemoradiotherapy compared with neoadjuvant radiotherapy was proven to be more effective in the downstaging of tumors (*p* < 0.001) and was associated with lower invasion of perineurium, lymphatic, and venous vessels (*p* = 0.008). Additionally, administration of chemotherapy preoperatively (vs. postoperatively) was linked to higher treatment compliance (82.0% vs. 42.9%) [43]. In another study, published by Gérard JP et al., concerning 742 patients aged 75 years and below with a WHO performance status of 0–1, neoadjuvant chemoradiation was compared with neoadjuvant radiotherapy alone in T3-T4 and M0 rectal cancers. It was shown that nCRT had a higher pCR (pathologic complete response) rate (11.4% vs. 3.6%; *p* < 0.0001) and a lower 5-year cumulative local recurrence rate (8.1% vs. 16.5%; *p* = 0.004). The study revealed no difference in 5-year overall survival and 5-year progression-free survival [44].

Nassoiy et al., in 2022, retrospectively analyzed 3868 locally advanced rectal cancer patients aged ≥80 years. They were grouped depending on treatment type: (1) surgery + adjuvant chemotherapy or radiotherapy (n = 404), (2) surgery alone (n = 1191), and (3) nCRT + surgery (n = 2273). The nCRT group achieved higher rates of R0 resection (92.4% vs. 79.7% vs. 87.1%; nCRT + surgery vs. surgery + adjuvant treatment vs. surgery alone, respectively) and lower rates of resection margin involvement and perineural invasion compared to the other groups. Neoadjuvant chemoradiotherapy was significantly associated with a greater likelihood of an R0 (aOR = 2.16, 95% CI, 1.62–2.88). Those who obtained the margin-negative resection (R0) had higher median OS in comparison with those who did not (4.39 years [95% CI, 4.19–4.58] vs. 2.04 [95% CI, 1.70–2.40]; R0 vs. non-R0, respectively). Receiving neoadjuvant chemoradiotherapy + surgery, compared with surgery + adjuvant chemotherapy/radiotherapy, resulted in higher median survival (5.11 years [95% CI, 4.90–5.41] vs. 4.19 years [95% CI, 3.78–4.91], respectively). Additionally, nCRT was independently associated with a 25% decreased risk of death [45].

### 4.3. Complications and Limitations of Standard Treatment

Rectal cancer treatment seems to be still evolving. Despite apparent improvements in treatment, there is an ongoing debate about the applicability of the standard approach to all patients. This section focuses on the complexities and outcomes of the standard treatment, particularly emphasizing its complications and limitations among older patients.

In recent decades, despite prominent advances in oncological treatment and improved short-term and long-term survival of older adults diagnosed with rectal cancer [46], there remains doubt as to whether the standard treatment is a one-size-fits-all approach. The treatment entails a risk of various complications and side effects. Regarding chemotherapy, these may include early and late toxicity, peripheral neuropathy, diarrhea, fatigue, mucositis, and neutropenia [39,47,48]. Patients undergoing radiotherapy may suffer from skin ulceration, radiation-induced proctitis, local pain, or marrow suppression [39]. Surgical treatment is potentially associated with blood loss, urinary dysfunction, wound infection and dehiscence, anastomotic leakage, or a longer stay at the hospital [39,49].

De Felice et al. enrolled 20 elderly (≥70 years) patients treated for stage IIa-IIIc rectal cancer to describe tolerance and outcomes of nCRT. The study demonstrated gastrointestinal toxicity (especially proctitis and diarrhea) to be the most common acute side effect related to neoadjuvant chemoradiotherapy. The second finding was that patients who had scores >1, assessed by using the ACE-27 (adult comorbidity evaluation-27), more often developed acute side effects (100% vs. 22.2%, *p* = 0.015); ACE-27 > 0 had no impact on OS (overall survival) and DFS (disease-free survival) [50]. Rosa et al., in their study concerning 117 patients with LARC who were ≥70 years old, demonstrated that nCRT was most often associated with toxicity of the lower gastrointestinal tract (recorded in 80 patients; 68.4%) as an acute side effect of this treatment. Other acute side effects included skin toxicities (n = 54; 46.2%), genitourinary toxicities (n = 29; 24.8%), and hematologic toxicities. Furthermore, patients who reached a pCR or a near-to-pCR were observed to have significantly better OS, 3-year DFS, and 5-year DFS compared to the patients who did not reach these outcomes [51].

## 5. “Watch-and-Wait” Strategy in Rectal Cancer

The treatment of LARC in frail patients is the subject of growing attention. The problem of obtaining satisfactory cancer treatment outcomes with acceptable risks and complications of invasive procedures has led to the development of alternative approaches. The following section discusses an emerging modality, the “watch-and-wait” strategy, with a focus on frail cancer patients. We highlight the importance of tailoring rectal cancer therapy to functional age.

In opposition to the standard treatment (nCRT + TME + adjuvant chemotherapy) in locally advanced rectal cancer, the experts’ panel of 2021, the SICG (Italian Society of Geriatric Surgery), the SIFIPAC (Italian Society of Surgical Pathophysiology), the SICE (Italian Society of Endoscopic Surgery and New Technologies), and the WSES (World Society of Emergency Surgery) consensus recommended short-course radiotherapy with delayed (>4 weeks) surgery as a treatment of LARC in frail elderly patients (evidence IIB) [41]. There also emerged a new, alternative approach focused on surgical therapy de-escalation in a carefully chosen group of patients. The concept of a “watch-and-wait” strategy assumes that surgical procedure and adjuvant chemotherapy might be omitted if the complete clinical response (cCR), no evidence of primary tumor assessed by radiological and endoscopic imaging, is reached after chemoradiotherapy or radiotherapy alone [10,31,52,53]. The “watch-and-wait” strategy realizes strictly scheduled and precise surveillance of local recurrence utilizing digital rectal examination, endoscopy, and MRI [52]. Clinicians who support this approach underline its ability to preserve sphincter functions and abandon stoma creation related to surgery [52,54,55]. On the other hand, implementation of this strategy may risk local regrowth and overlooking microscopic portions of the tumor, but once tumor tissue is observed, treatment may be complemented [52,53,55].

The group of patients diagnosed with rectal cancer consists of a heterogeneous population of fit patients without comorbidities, as well as those frail and vulnerable. More and more authors highlight the importance of adjusting cancer treatment not solely to chronological age, but rather to functional age and capability [56]. Francesca De Felice et al. proposed an algorithm for LARC treatment in a group of elderly patients. The entire group was divided into three subgroups—fit, vulnerable, and frail—based on the patients’ health evaluation (incl. ACE-27, MNA, Mini-Cog, and G8). The article and the algorithm highlighted the fact that the so-called “standard treatment modality” might not be suitable for frail patients. They suggested that for the aforementioned group, radiotherapy alone or palliative therapy should be chosen as a treatment method. The authors also proposed a “watch-and-wait” policy as an option for vulnerable patients who completed CRT and achieved a complete clinical response after 8 weeks [10].

TME has become the standard approach for rectal cancer resection. Rutten et al. suggested that TME surgery might be beneficial for younger patients diagnosed with rectal cancer. However, the authors noted that studies that compared mortality and outcomes of rectal cancer treatment before and after the introduction of this procedure are based on the general population, mainly consisting of younger patients. The article points out the underrepresentation of the elderly in clinical studies and states that this standard approach should not be simply adopted for elderly patients, as mortality after the introduction of TME as a standard cancer resection did not significantly decrease in older patients [57]. Since nCRT enables us to obtain low local regrowth rates of rectal cancer and good local control of the disease as a result, there more often comes up the question of whether a standard modality (nCRT + surgery + adjuvant chemotherapy) should be proposed for frail patients. Habrgama and colleagues in a study from 2005 enrolled 260 patients diagnosed with resectable distal rectal cancer. Regardless of their pretreatment clinical staging, all of them were initially treated with nCRT (5-FU, leucovorin, 50.4 Gy). After 8 weeks from chemoradiation completion, patients were re-staged (through physical examination, digital rectal examination, CEA serum level, proctoscopy, radiography, and CT scans) and assigned either to a complete clinical response group or an incomplete clinical response group. In total, 71 patients (28%; mean age 58.1, range 35–92 years) had no evidence of cancer (cCR) after completion of nCRT, and they were observed by strictly scheduled follow-up visits. At the time of cancer diagnosis, 69% of them presented with T3 rectal cancer, and 11.3% had T4 disease. In 22.5% (16 patients), node involvement was observed (N+). On the other side, the rest (189 patients; 72%) were re-staged as incomplete CR, and they had a surgical intervention. With a mean follow-up of 57.3 months (range 18–156 months), in the cCR group, only two patients (2.8%) presented endorectal recurrence (after 56 and 64 months; both successfully treated with salvage brachytherapy and transanal excision), and three patients (4.2%) were diagnosed with systemic metastases. Moreover, no deaths due to cancer were observed. The 5-year cancer-related overall survival and 5-year disease-free survival rates were 100% and 92%, respectively [58].

We cannot ignore the premise that surgery is generally associated with high chances of LARC cure, but at the same time, we must emphasize that in older patients (especially frail ones), whose expected lifetime is lower than in young, non-frail patients, the other approach may be appropriate [14,31]. There is a high chance that frail patients diagnosed with rectal cancer will benefit from omitting the TME procedure since they may suffer high morbidity and mortality related to surgery, and their predicted lifetime is generally low. They may not outlive potential rectal cancer progression and can avoid quality of life decline after the surgical procedure and complications related to TME. Shorter treatment, fewer expected complications, fewer medical procedures, and a lower chance of institutionalization may be chosen by frail patients as a preference and may outperform the effects offered by surgery.

## 6. Emerging Need for Further Research

More and more often, the underrepresentation of older and frail patients in clinical trials is emphasized in the oncological literature [11,15,19,57,59]. The lack of clear data on treatment outcomes for frail patients may lead to overtreatment or undertreatment in individual cases. This problem may raise doubts about whether all elderly patients benefit from the “standard treatment” approach. The main reason for the insufficient representation in clinical studies is probably the difficulty to clearly and universally determine the study population of frail patients since the group of rectal cancer patients is heterogeneous and different approaches to frailty syndrome exist. The other reason may be the way that inclusion and exclusion criteria are formulated for clinical trials. They exclude patients less resilient to treatment and those with certain comorbidities common among frail patients [11,57,60].

The adoption of a “watch-and-wait” strategy after neoadjuvant chemoradiotherapy is based on cCR that is detected by digital rectal examination, endoscopic images, MRI, and PET-CT scans [52,61]. As studies revealed that cCR does not fully correlate to pathologic complete response (pCR), assessed through pathological examination of a surgical specimen, novel tools are needed to more precisely state the no-evidence of cancer in the clinical setting [61,62]. One of the paths to a better understanding of rectal cancer response to neoadjuvant treatment might be research on miRNA, cell-free DNA (cfDNA), circulating tumor DNA (ctDNA), and circulating tumor cells (CTCs) detected using a non-invasive diagnostic tool, liquid biopsy. Recent papers described promising results of the analysis and monitoring of these molecules in the context of predicting response to neoadjuvant chemoradiotherapy [63,64,65,66,67,68,69].

As today’s world population is aging, frailty syndrome incidence rises with age, and older adults are most commonly diagnosed with rectal cancer, there is a discussion about whether frailty screening should be a “standard of care” on oncologic wards [6,13,19,59,70]. A universal screening tool devised by interdisciplinary research teams will standardize the group of frail patients in clinical research and improve patients’ daily care through more individualized decisions in the treatment [19,56]. We emphasize the emerging and urgent need for new multicenter clinical trials that will include a higher fraction of elderly, frail patients. The inclusion and exclusion criteria should be properly assessed and determined by a multidisciplinary team (comprising geriatricians and oncologists), so they can encompass a greater number of frail rectal cancer patients and thus facilitate personalization of care in this group of patients.

**Figure 2:**
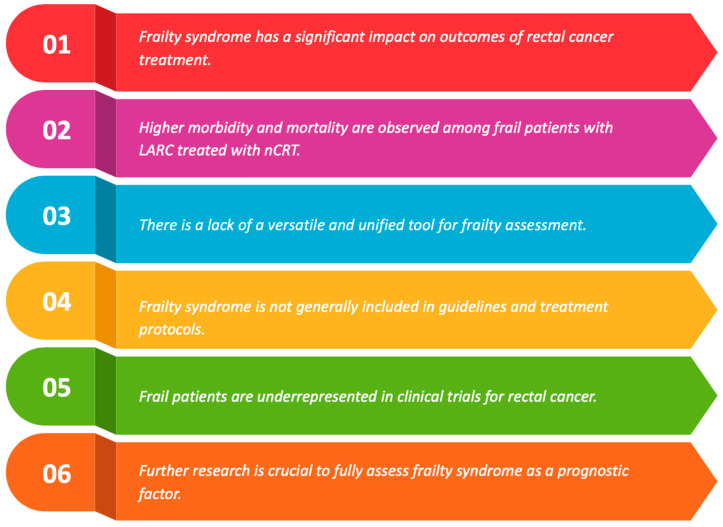
Frailty syndrome and rectal cancer—current challenges and future directions.

Figure 2 presents the ongoing challenges and research directions regarding frailty syndrome.

## 7. Conclusions

Rectal cancer remains a prominent oncological disease worldwide, and despite certain advances, its treatment presents a real challenge for some groups of patients. The general population is aging, which will directly impact the prevalence rate of frailty syndrome. This particular deterioration of one’s health may be reversed; hence, efforts should be made to introduce routine, standardized screening of hospitalized patients. The characteristics of frailty syndrome as a health status should prompt medical experts and specialists in various fields to make special adjustments to guidelines and treatment algorithms so that all individualities of senectitude are accounted for. In our view, it can be performed as long as clinical trials include a higher portion of precisely described and standardized groups of frail patients. The treatment of a frail person should be planned by a multidisciplinary, trained team consisting of a geriatrician, oncologist, surgeon, anesthesiologist, dietician, and physical therapist.
